# New Antimicrobial Bromotyrosine Analogues from the Sponge *Pseudoceratina purpurea* and Its Predator *Tylodina corticalis*

**DOI:** 10.3390/md13031389

**Published:** 2015-03-16

**Authors:** Michael P. Gotsbacher, Peter Karuso

**Affiliations:** Department of Chemistry & Biomolecular Sciences, Macquarie University, Sydney, NSW 2109, Australia; E-Mail: michael.gotsbacher@sydney.edu.au

**Keywords:** alkaloid, bromotyrosine, *Pseudoceratina*, sponge, Tylodina, antibiotics, marine natural products

## Abstract

Bioassay-guided fractionation of extracts from temperate Australian collections of the marine sponge *Pseudoceratina purpurea* resulted in the isolation and characterisation of two new and six known bromotyrosine-derived alkaloids with antibiotic activity. Surprisingly, a single specimen of the mollusc *Tylodina corticalis*, which was collected while feeding on *P. purpurea*, contained only a few of the compounds found in the sponge suggesting selective accumulation and chemical modification of sponge metabolites.

## 1. Introduction

Sponges of the order Verongida are unique in the Demospongiae as the only group that contains alkaloids derived from bromotyrosine and sterols of the aplystane type; these are distinct chemotaxonomic markers for all the verongid genera [1,2,3]. The alkaloids have been reported to have antimicrobial [4,5,6,7], antifungal [8,9], cytotoxic [10,11,12,13,14,15,16,17,18,19,20,21], and antimalarial [22,23,24] activity for example and many have unique modes of action. Consequently, verongid sponges have no natural predators other than a few specialised molluscs such as nudibranchs [25].

In our continuing effort to isolate and identify new drug candidates from marine sponges [26] we screened temperate water sponges for antimicrobial and herbicidal activity. This revealed that the ethyl acetate partition of the ethanolic extract of *Pseudoceratina purpurea* (Carter 1880) displayed selective activity against *Staphylococcus aureus* (35 mm zone of inhibition in a disc diffusion assay with no activity against *P. aeruginosa*). This sponge has previously been reported to be a rich source of bromotyrosine-derived alkaloids with wide ranges of biological activity [8,12,16,27,28,29,30,31,32,33,34,35]. However, temperate Australian specimens of this sponge have not previously been investigated and it is a characteristic of this species that different alkaloid profiles have been found from sponges collected at different locations.

In addition we were also encouraged to investigate the natural products of this sponge because we observed, and collected, a single specimen of the opisthobranch *Tylodina corticalis* (Tate, 1889) feeding on this sponge. As *Tylodina* is a partially shelled mollusc it would be interesting to determine if this mollusc accumulates defensive chemicals from its diet, lending weight to the theory that chemical defence is a pre-adaptation to loss of the protective shell found in most molluscs [25,36]. *Tylodina corticalis* has not previously been chemically investigated, but Proksch *et al.* have published their work on *Tylodina perversa* from the Mediterranean [5,37,38,39]. In their study, they found that *T. perversa*, fed on a diet of *Aplysina aerophoba* sequestering uranidine, isofistularin-3, aerophobin 1, aerophobin 2, and aplysinamisin-1. Whereas aerophobin 2 and isofistularin-3 made up about one third of the total alkaloids each in the sponge, aerophobin 2 constituted ~70% of the alkaloids found in the mantles, mucus, and egg masses of *T. perversa*, indicating selective sequestration by the mollusc. Another 20% of the alkaloids found in *T. perversa* was attributed to aerothionin, which was not detected in the prey sponge suggesting that this compound may have come from a previous diet of a related sponge (e.g., *A. cavernicola*). Several species of *Aplysina* have been reported to contain high concentrations of aerothionin [13,40,41,42,43].

Herein we report the identification of 16 bromotyrosine alkaloids from *Pseudoceratina purpurea* and *Tylodina corticalis* by high resolution LC-MS/UV-Vis spectroscopy. Eight of these were isolated preparatively and subject to 2D NMR analysis, revealing two to be new compounds.

## 2. Results and Discussion

**Figure 1:**
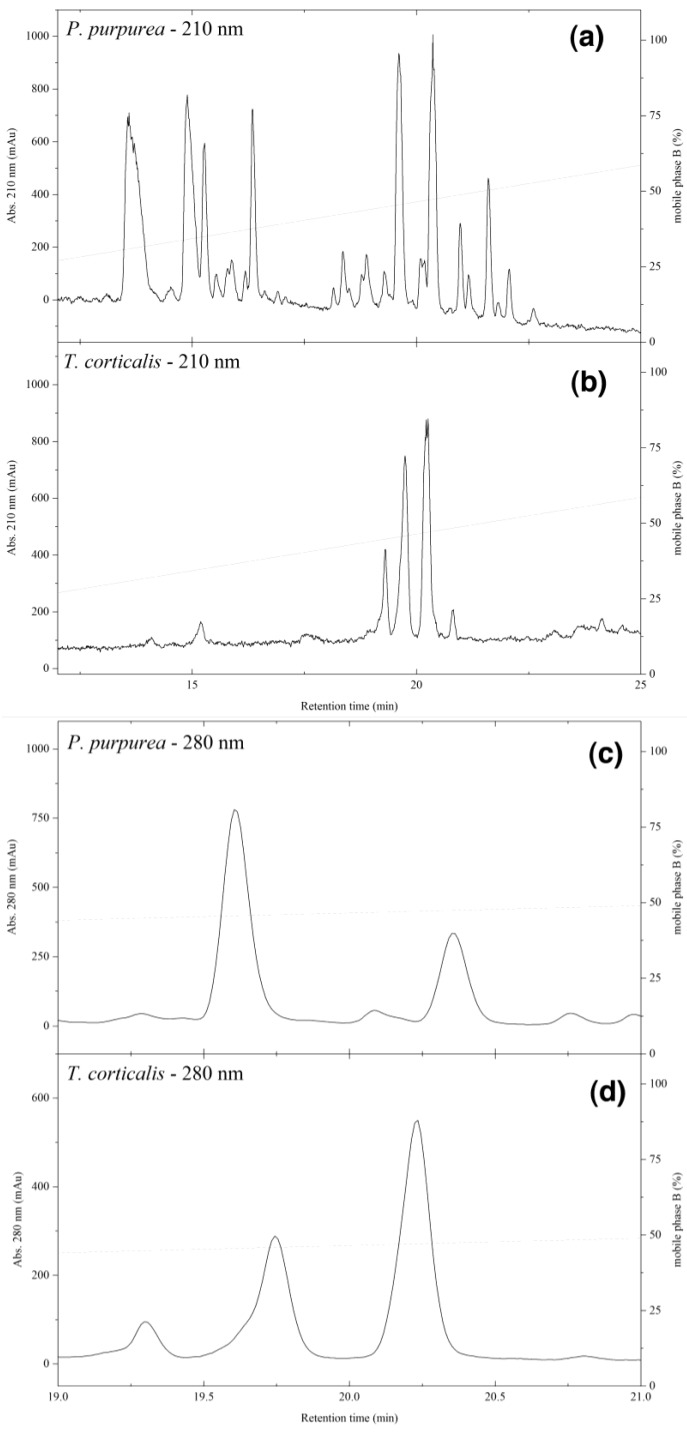
Low resolution-LCMS chromatograms (λ_max_ 210 nm) of ethyl acetate partitions of *P. purpurea* (**A**) and *T. corticalis* (**B**); Panels (**C**) and (**D**) are expansions of the 19–21 min regions (λ_max_ 280 nm).

The ethanol extracts of both the sponge and mollusc appeared as yellow solutions, which quickly turned dark-purple when exposed to air. This suggested the presence of the well-known verongid sponge pigment uranidine [44]. As the crude extract showed selective activity against *S. aureus* (Supplementary Information), activity against this organism was used for the bioassay-guided isolation. The ethyl acetate partition of the ethanolic extract of *P. purpurea* was subject to gel filtration (Sephadex LH-20) and fractions (9–22), with activity against *S. aureus*, were combined. A similar extraction of *T. corticalis* yielded only 12 mg in the ethyl acetate partition so this was not subject to gel filtration but analysed without further fractionation. Comparison of the two extracts (Figure 1A,B) suggested a similar pattern of metabolites eluting around 20 min but the *T. corticalis* extract was much simpler in composition. However, more careful examination of this region (Figure 1C,D) showed that even the major peaks of the sponge and mollusc did not overlap exactly. Using nanospray HRMS in combination with UV-Vis spectra (Figure S1; Supplementary Information) allowed us to tentatively identify most of the compounds from both organisms (Table 1). While both organisms contained bromotyrosine-derived alkaloids, very little overlap in secondary metabolites between the two organisms existed (highlighted in grey in Table 1), which was surprising considering there was evidence of extensive feeding by *T. corticalis* on the sponge sample that was collected and extracted in this work.

**Table 1:** Low and high resolution LC-MS data from extracts of *P. purpurea* and *T. corticalis*.

R_T_ (min) ^a^	λ_max_ (nm)	Br*_x_*^b^	HR *m/z*	Molecular Formula [M + H]^+^	Δppm	*P. purpurea*^c^	*T. corticalis*^c^	Compound ^c^
13.7–14.2	226, 286	2	489.9717, 491.9689, 493.9665	C_15_H_18_Br_2_N_5_O_4_	−0.306, −1.059, −1.413	+++	+	pseudoceratinin A (**3**)
14.67	232, 286	2	494.0030, 496.0004, 497.9983	C_15_H_22_Br_2_N_5_O_4_	−0.306, −0.859, −0.913	+	+	purealidin L (**10**)
15.1–15.4	228, 285	2	503.9878, 505.9848, 507.9824	C_16_H_20_Br_2_N_5_O_4_	+0.144, −0.809, −1.163	+++	++	aerophobin 2 (**4**)
15.41	234, 276, 340	2	680.9950, 682.9920, 684.9897	C_24_H_23_Br_2_N_6_O_8_	+0.937, +0.183, −0.070	+		ceratinadin B (**11**)
**16.35**	**234, 276, 337**	**2**	**695.0095, 697.0067, 699.0046**	**C_25_H_25_Br_2_N_6_O_8_**	**−0.013, −0.767, −0.820**	**++**		**new (ceratinadin D) (1)**
18.48	209, 286	2	489.9722, 491.9692, 493.9668	C_15_H_18_N_5_O_4_Br_2_	+0.194, −0.759, −1.113	+		purealidin M (**12**)
19.00	209, 286	2	503.9878, 505.9857, 507.9835	C_16_H_20_Br_2_N_5_O_4_	+ 0.144, +0.091, −0.063	+		aerophobin 2 isomer
19.41	210	0	293.2335	-	-	+	++	C18 fatty acid methyl ester?
19.3–19.8	223, 280	4	713.8461, 715.8431, 717.8400, 719.8376, 721.8355	C_21_H_24_Br_4_N_3_O_5_	+ 1.502, +0.749, −0.305, −0.658, −0.711	+++	+	hexadellin A (**4**)
19.85	203, 280	4	741.8751, 743.8729, 745.8709, 747.8689, 749.8669	C_23_H_28_Br_4_N_3_O_5_	−0.598, −0.751, −0.705, −0.658, −0.611	~	+++	purealidin P (**13**) or Q (**14**)
20.08	210	0	293.2333	-	-	~	~	C18 fatty acid methyl ester?
20.13	203, 280	4	757.8720, 759.8688, 761.8660, 763.8634, 765.8616	C_23_H_28_Br_4_N_3_O_6_	+1.388, +0.234, −0.519, −1.073, −0.826		+++	purealidin T (**15**) or purealidin P *N*-oxide (**16**)
20.30	impure	3	633.9558, 635.9530, 637.9503, 639.9482	C_22_H_27_Br_3_N_3_O_4_	+1.179, +0.426, −0.227, −0.281	+		aplysamine 7 (**17**), purpurealidin H (**18**), purpuramine I (**19**) or purpuramine L (**20**)
20.47–20.53	222, 280	3	647.9707, 649.9673, 651.9644, 653.9627	C_23_H_29_Br_3_N_3_O_4_	+0.429, −0.924, −1.777, −1.431	+++	+	aplysamine 2 (**6**)
20.90	234, 279	3	663.9649, 665.9630, 667.9609, 669.9587	C_23_H_29_Br_3_N_3_O_5_	−0.285, −0.139, −0.192, −0.345		++	purpuramine J (**21** ) (aplysamine 2 *N*-oxide)
20.98	*210, 278*	3	619.9397, 621.9368, 623.9340, 625.9320	C_21_H_25_Br_3_N_3_O_4_	+ 0.729, −0.124, −0.877, −0.831	++		16-debromo-aplysamine 4 (**7**)
**21.71**	***209, 278***	**4**	**697.8505, 699.8476, 701.8446, 703.8420, 705.8401**	**C_21_H_24_Br_4_N_3_O_4_**	**+1.017, +0.163, −0.790, −1.343, −1.197**	**++**		**new (aplysamine 8) (2)**
22.25	*209, 278*	4	879.8942, 881.8915, 883.8894, 885.8871, 887.8850	C_27_H_30_Br_4_N_7_O_7_	+ 0.727, +0.073, +0.020, −0.233, −0.287	++		purealine (**8**)
24.27	240, 308, 363	2	404.9809, 406.9787, 408.9765	C_14_H_19_Br_2_N_2_O_2_	+ 0.120, −0.033, −0.186		+	new unknown

^a^ Retention times from LR LCMS trace (Figure 1); ^b^ number of bromine atoms from LR-MS molecular ion isotopic pattern; ^c^ +++; major metabolite, ++; medium abundance metabolite, +; minor metabolite; - no molecular formula was found and ~; trace metabolite; ^d^ tentative assignment based on HR-MS and UV (see Figure 2 and Figure 7); Grey indicates similarities between *P. purpurea* and *T. corticalis. Italic* indicates low accuracy for UV spectrum due to low sample concentration. **Bold** indicates new compounds.

**Figure 2:**
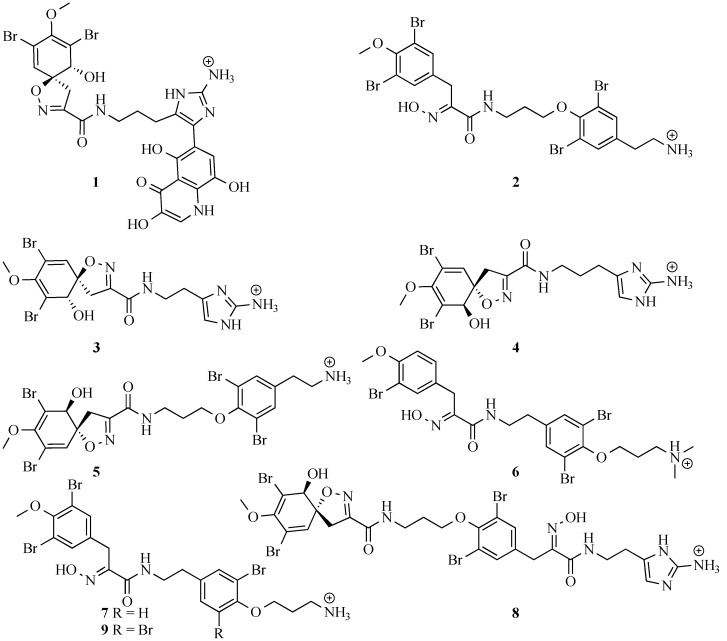
Bromotyrosine-alkaloids isolated from the marine sponge *P. purpurea*.

Preparative HPLC separation of the eight major compounds from the sponge confirmed the tentative structures assigned by HR LC-MS and UV-Vis and that two of the compounds (**1** and **2**) were new (Figure 2). The six known compounds were (−)-pseudoceratinine A (**3**) [45]; (+)-aerophobin 2 (**4**) [46]; (−)-hexadellin A (**5**) [47]; aplysamine 2 (**6**) [48]; 16-debromoaplysamine 4 (**7**) [49]; (−)-purealin (**8**) [50] (Figure 2) and their NMR spectral data matched those reported in the literature. The two new compounds were subject to full spectral analysis to determine their structure and stereochemistry.

Ceratinadin D (**1**) was obtained as an optically active solid ( ${\left[\text{α}\right]}_{\text{D}}^{20}$ +52) with UV maxima at 234, 276, and 337 nm, suggesting the presence of a spiro-cyclohexadienyl-isoxazoline (λ_max_ 234, 276 nm) and a uranidine moiety (λ_max_ 337 nm). The IR absorptions indicated the existence of OH and/or NH (3420, 3380, and 3290 cm^−1^) and an amide (1677 cm^−1^). The low resolution mass spectrum displayed an isotopic cluster (694.95, 696.95, 699.00 amu in ratio 1:2:1) suggesting two bromines were present. High resolution mass spectrometry suggested a molecular formula C_25_H_24_^79^Br_2_N_6_O_8_ (0.013 ppm error). These data matched reasonably well with those reported for (+)-ceratinadins A and B [9].

The ^13^C NMR spectrum indicated the presence of 25 carbons (Table 2), one *O*-methyl (δ 59.7), four methylenes (δ 39.0, 38.2, 28.1, 21.6), three sp^2^ methines (δ 131.2, 124.2, 113.7), one sp^3^ methine (δ 73.5), 13 sp^2^ quaternaries (δ 154.5, 149.6, 147.6, 146.6, 139.9, 137.4, 128.9, 122.4,120.9, 117.8, 113.1, 112.6, 103.2), one sp^3^ quaternary (δ 90.1) and two carbonyls (δ 173.2, 158.9).

**Table 2:** NMR (DMSO-*d*_6_, 600 MHz) data for new bromotyrosine compound (**1**).

Position	δ_C_	Type	δ_H_, m (*J* in Hz)	COSY (H no.)	^1^H-^13^C HMBC (C no.)
1	73.5	CH	3.90 d (7.7)	9	3, 4, 5, 6
2	120.9	C	-		
3	147.6	C	-		
4	113.1	C	-		
5	131.2	CH	6.54 s	1	2, 3, 4, 6 (w) ^a^, 10
6	90.1	C	-		
8	59.7	CH_3_	3.63 s		3
9	-	OH	6.36 d (7.7)	1	1 (w), 4 (w), 6 (w)
10	39.0	CH_2_	3.59 d (3.6), 3.15 d (3.6)	10	1, 5, 6, 11
11	154.5	C	-		
14	158.9	C	-		
16	-	NH	8.51 t (5.8)	17	14
17	38.2	CH_2_	3.13 m	18	18, 19
18	28.1	CH_2_	1.73 m	17, 19	17, 19
19	21.6	CH_2_	2.51 m	18	18 (w), 20, 24
20	122.4	C	-		
21	-	NH	11.93 bs	23	20 (w)
22	146.6	C	-		
23	-	NH	12.15 bs	21	-
24	117.8	C	-		
25	-	NH_2_	7.28 bs		
1′	-	NH	11.76 d (6.4)	2′	3′, 5′
2′	124.2	CH	7.64 d (6.4)	1′	4′, 10′
3′	139.9	C	-		
4′	173.2	C	-		
5′	112.6	C	-		
6′	149.6	C	-		
7′	103.2	C	-		
8′	113.7	CH	6.83 s		24, 6′, 9′, 10′
9′	137.4	C	-		
10′	128.9	C	-		
11′	-	OH	8.93 bs		
12′	-	OH	14.3 bs		5′, 6′ (w)
13′	-	OH	10.25 bs		10′

^a^ (w) denotes a weak correlation.

**Figure 3:**
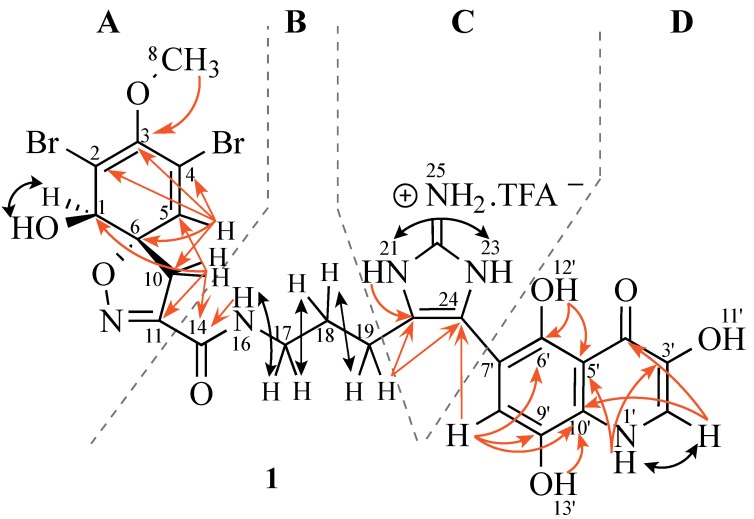
Structure and selected COSY (black) and HMBC (red) correlations of new bromotyrosine compound **1**.

Use of one and two-dimensional NMR data (Table 2) enabled the construction of four substructures (Figure 3). Inspection of ^1^H, ^13^C, and ^1^H-^1^H COSY NMR spectra suggested that the following proton signals belonged to the same spin system: δ_H_ 6.54 (sp^2^ methine), δ_H_ 3.90 (sp^3^ methine), δ_H_ 3.63 (OMe), and an AB system (δ_H_ 3.59 and δ_H_ 3.15 each 1H) characteristic of a spirocycloisoxazoline ring previously published from other verongid compounds [51]. HMBC correlations from H-5 to C-1, C-2, C-3, H-8 to C-3, and H-10a/b to C-1, C-6, C-11 confirmed the 1-hydroxy-2,4-dibromo-3-methoxy-11-carbonyl spirocyclohexadienyl isoxazole (substructure **A**) and the connection between substructures **A** and **B** was obtained by an HMBC correlation from H-10a/b to C-14.

The signal at δ_H_ 8.51 (NH) showed a coupling to δ_H_ 3.13 (H-17) and an HMBC correlation to the amide carbonyl (C-14; δ 158.9). Further COSY correlations from H-17 to H-18 (δ_H_ 1.73), and in turn H-19 (δ_H_ 2.51) supported the conclusion that substructure **B** was an amide unit connected to a propyl chain (*c.f.* an ethyl chain in ceratinadin A and B).

**Figure 4:**
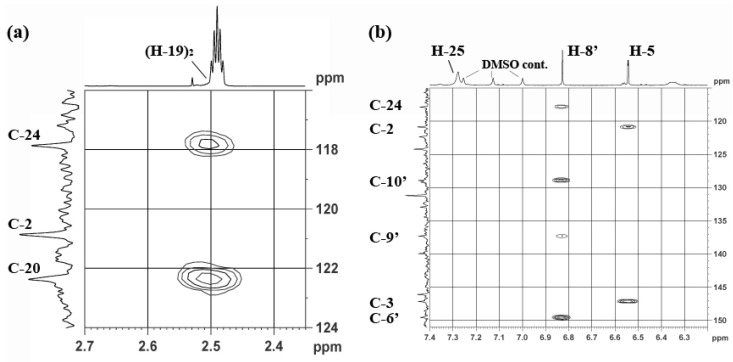
Selected HMBC correlations of **1**: (**a**) from H-19 (δ 2.51) to C-20 (δ 122.4) and C-24 (δ 117.8) of the imidazole moiety. H-19 lies under the large DMSO peak (2.49 ppm). (**b**) from H-8′ (δ 6.83) ^3^*J* couplings to C-24 (δ 117.8) of the imidazole moiety, and to C-6′ (δ 149.6), C-10′ (δ 128.9) and a ^2^*J* coupling to C-9′ (δ 137.4) of the uranidine moiety.

HMBC correlations between H-19 and C-20 and C-24 (Figure 4) indicated that the CH_2_ at δ_C_ 21.2 ppm was connected to the imidazole ring. The w-COSY correlation between two broad singlets at δ_H_ 11.93 (N-21H) and δ_H_ 12.15 (N-23H), and the 2H singlet at δ_H_ 7.77 suggested the imidazole is present in the form of an imidazolium salt. The presence of a homohistamine unit is commonly observed in verongid metabolites [51] and was also found in other metabolites from this sponge. For example, aerophobin, pseudoceratinin A, and purealin show similar NMR data for this substructure and provided good evidence for unit **B**/**C**. Although there were no correlations to C-22, a quaternary carbon at δ 146.6 was observed, which matches literature values for 2-aminoimidazole. Because of resonance and long T1 relaxation times, it is common not to observe correlations with guanidine carbons.

The HMBC correlations in the trihydroxy quinolinone (uranidine) ring system (H-1′ to C-2′ and C-10′) matched those previously reported for 7-substituted 3,6,9-trihydroxy quinolinone [52]. In addition, H-8′ also showed a correlation to C-10′ (see Figure 4b), which could only be observed with 7-substitution (*c.f.* from H7′ a ^4^*J*_CH_ coupling would not be observable). If the uranidine moiety was attached at C-8′; ^3^*J_CH_* couplings should be observed from H-7′ to C-9′ and C-5′. The site of attachment at the imidazole was clearly indicated by strong correlations between C-24 and H-19 and H-8′ (Figure 4). Further evidence for the presence of unit **D** arose from ^1^H-^1^H coupling between H-2′ (δ_H_ 7.64, d, *J* = 5.8 Hz) and H-1′ (δ_H_ 11.76, d, *J* = 5.8 Hz).

The chemical shifts for subunits **A**, **C** and **D** all aligned very well with those described for ceratinadin B [9], a compound similar to the one described here. However, ceratinadin B contains a histamine unit instead of homohistamine (subunit **B**) and thus a lower molecular weight. Due to the similarity of these two compounds, and with the other ceratinadins, we named this compound ceratinadin D. Finally, the specific rotation of **1** is very similar to that of ceratinadin B strongly suggesting the same absolute stereochemistry (1*R*, 6*S*).

Aplysamine 8 (**2**) was a simple bromotyrosine derivative (λ_max_ 278 nm) with four bromines (M + H pentet; *m/z* 698 (1), 700 (4), 702 (6), 704 (4), 706 (1)). The HRMS was consistent with a molecular formula C_21_H_23_Br_4_N_3_O_4_ (Table 1). In addition, three characteristic bands in the infrared spectrum (ν_max_ 3572, 1672 and 1001 cm^−1^), confirmed the presence of an oxime.

These data suggested the compound was aplysamine 4 (**9**) [10]. However, comparison of the ^1^H-NMR spectrum reported for aplysamine 4 differed to our compound (Table 3) in the following ways: In aplysamine 4, the aromatic protons from ring A and B are all at δ_H_ ~7.44. We found two different environments for the aromatic ring protons, where H-2 were at δ_H_ 7.44 but H-20 were at 7.55. This could be attributed to the use of different solvents but what was also different was the chemical shift of the ethyl and propyl-chain carbons. In aplysamine 4 the propyl group terminated with an ammonium ion (C-20 = 39.0 ppm) whereas in our compound, this carbon was at 36.2 ppm. Similarly the carbon shifts for the ethyl group did not match those of aplysamine 4.

**Table 3:** NMR (DMSO-*d*_6_, 600 MHz) data for new bromotyrosine aplysamine 8 (**2**).

Position	δ_C_	Type	δ_H_, m ( *J* in Hz)	COSY (H no.)	^1^H-^13^C HMBC (C no.)	ROESY (H no.)
1	136.3	C	-			
2	132.9	CH	7.44 s	7	3, 4	7
3	117.1	C	-			
4	151.8	C	-			
6	60.4	CH_3_	3.75 s		4	
7	27.9	CH_2_	3.76 s	2	1, 2, 8, 11	2
8	151.0	C	-			
10	-	OH	12.02 s		8 (w) ^a^	
11	163.0	C	-			
13	-	NH	8.12 t (6.0)	14	14	7, 14, 16, 15 (w)
14	36.2	CH_2_	3.38 m	13, 15	11, 15, 16	15, 16
15	29.6	CH_2_	1.96 m	14, 16	14, 16	13 (w), 14, 16
16	71.3	CH_2_	3.88 t (6.4)	15	14, 15	14, 15
18	151.3	C	-			
19	117.6	C	-			
20	133.2	CH	7.55 s		18, 19, 22	22, 23
21	136.8	C	-			
22	31.5	CH_2_	2.81 t (7.4)		20, 21, 23	20, 23
23	39.4	CH_2_	3.05 m	22, 24	21(w)	20, 22, 24
24	-	NH_3_	7.81 bs	23	-	23

^a^ (w) denotes a weak correlation.

**Figure 5:**
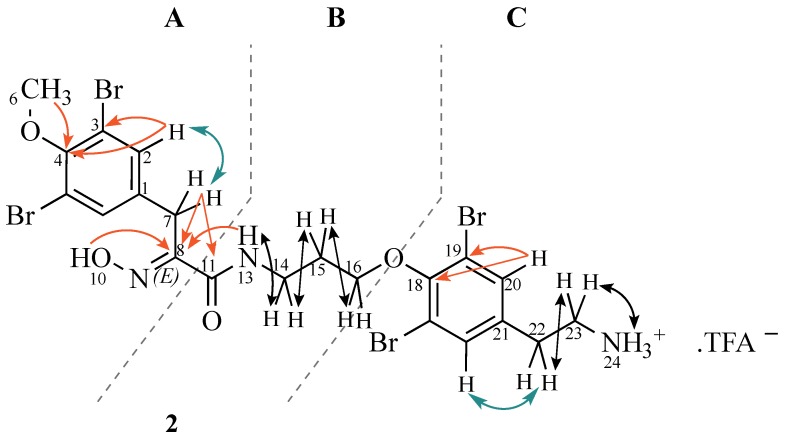
Structure and selected 2D-NMR correlations of new bromotyrosine compound (**2**). (Colours: red (HMBC), green (ROESY), black (COSY)).

The ^13^C NMR spectrum of our compound indicated the presence of 21 carbons, one *O*-methyl (δ 60.4), six methylenes (δ 71.3, 39.4, 36.2, 31.5, 29.6, 27.9), four sp^2^ methines (δ 133.2 × 2, 132.9 × 2), nine sp^2^ quaternary (δ 151.8, 151.3, 151.0, 136.8, 136.3, 117.6 × 2, 117.1 × 2) and one amide carbonyl (δ 163.0). All protonated carbons were assigned by HSQC experiment. Use of one and two-dimensional NMR data (Table 3) enabled the construction of three substructures (Figure 5). Inspection of ^1^H, ^13^C, and ^1^H-^1^H COSY NMR spectra suggested that the following proton signals belonged to substructure **A**: δ_H_ 12.02 (oxime), 7.44 (sp^2^ methine), 3.76 (sp^3^ methine) and 3.75 (OMe). HMBC correlations from H-2 to C-2, C-3, H-6 to C-4, and H-7a/b to C-8 and C-11 provided good evidence for the oxime-tyrosine unit **A**. The connection between substructures **A** and **B** was obtained by HMBC correlations from H-7a/b to carbonyl C-11 and from the amide proton H-13 to oxime carbon C-8. C-8 and C-11 could be easily distinguished by their typical chemical shifts as reported in the literature [51]. The geometry of the oxime was determined as *E* from the up-field ^13^C chemical shift of C-7 (δ_C_ 27.9), *c.f.* δ_C_ 35.7 for *Z*-oximes as observed in (*E, Z*)-*N,N′*-bis-[3-(3′-bromo-4′-hydroxyphenyl)-2-oximidopropionyl] cystamine [4].

**Figure 6:**
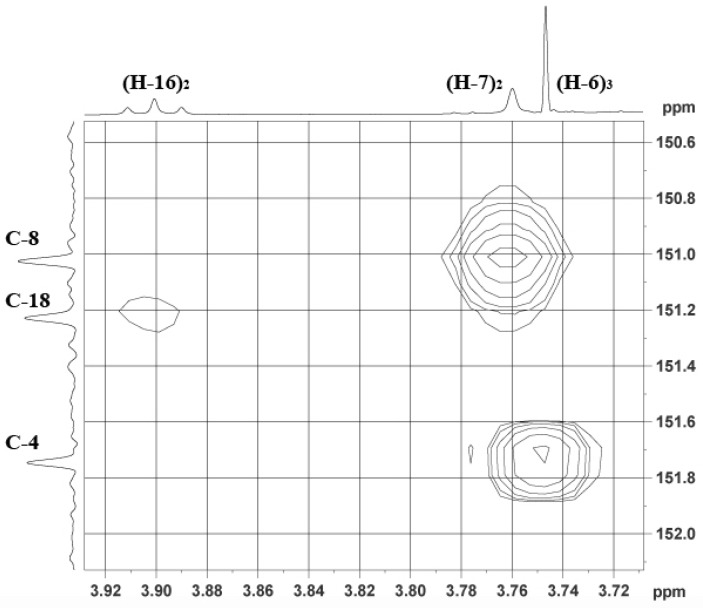
Selected HMBC correlations found in the unknown bromotyrosine (**2**; DMSO-*d*_6_, 600 MHz). A weak correlation between H-16 (δ 3.88) and C-18 (δ 151.3) indicates the link of substructures B and C.

The amide proton (δ_H_ 8.12) showed coupling to a methylene (δ_H_ 3.38) assigned to H-14. Further COSY correlations to δ_H_ 1.96, and then to δ_H_ 3.88 supported the assumption that substructure **B** was an amide unit connected to a propyl chain, attached to an oxygen (H-16 3.88 ppm; C-16 71.3 ppm from HSQC), which could only be explained by attachment to unit C via an ether linkage. Indeed, a weak correlation between C-18 (151.3 ppm) and H-16 was observed in the HMBC spectrum (Figure 6) and provided strong evidence for the assigned structure.

The signal at δ_H_ 7.55 (H-20) showed HMBC correlations to sp^2^ quaternary carbons (C-18, C-19) and a ROESY correlation to a methylene at δ_H_ 2.81/δ_C_ 31.5, consistent with attachment of an alkyl group directly to the aromatic ring, in support of substructure **C**. Direct coupling of δ_H_ 2.81 to δ_H_ 3.05, and of δ_H_ 3.05 to the primary amine protons at δ_H_ 7.80 were in good accordance to literature values [51] for a tyramine unit. Aplysamine 8 is thus an isomer of aplysamine 4 and considering the similarity in structure to aplysamines 1–7, no new name is required for this compound.

Putting the pieces together, we conclude that this is a new brominated tyrosine derivative (**2**) and, due to the similarities with previously described aplysamines 1–7, shall be named aplysamine 8.

Compounds **1**–**8** were bioassayed against *E. coli* and *S. aureus* (MTT microdilution assay; Supplementary Information). Only aplysamine 8 (**2**) was found to have any notable activity (MIC 125 μg/mL against *E. coli* and 31 μg/mL against *S. aureus*) *c.f.* ampicillin 1.1 μg/mL against *S. aureus*. Hexadellin (**6**), aplysamine 2 (**7**) and 16-debromoaplysamine 4 (**8**) had mild activity against *S. aureus* (125–250 μg/mL). While the MTT and disc diffusion assays are not directly comparable, the high antibacterial activity observed for the crude extract was not reflected in the bioassay of individual metabolites. This may be due to a cumulative effect of many weak antibiotics, synergistic or contingent effects as has previously been noted for other natural products [53].

Although opisthobranchs of the genus *Tylodina* are found in distant regions, they are exclusively associated with sponges of the order Verongida (in particular the family Aplysinellidae) [39]. *Tylodina corticalis* typically feed on the genus *Pseudoceratina* [54]. It has also been shown, that *Tylodina* spp accumulate the secondary metabolites (bromotyrosine alkaloids) from their prey, sequestered and utilised as feeding deterrents for themselves and their eggs [38,39,55,56].

**Figure 7:**
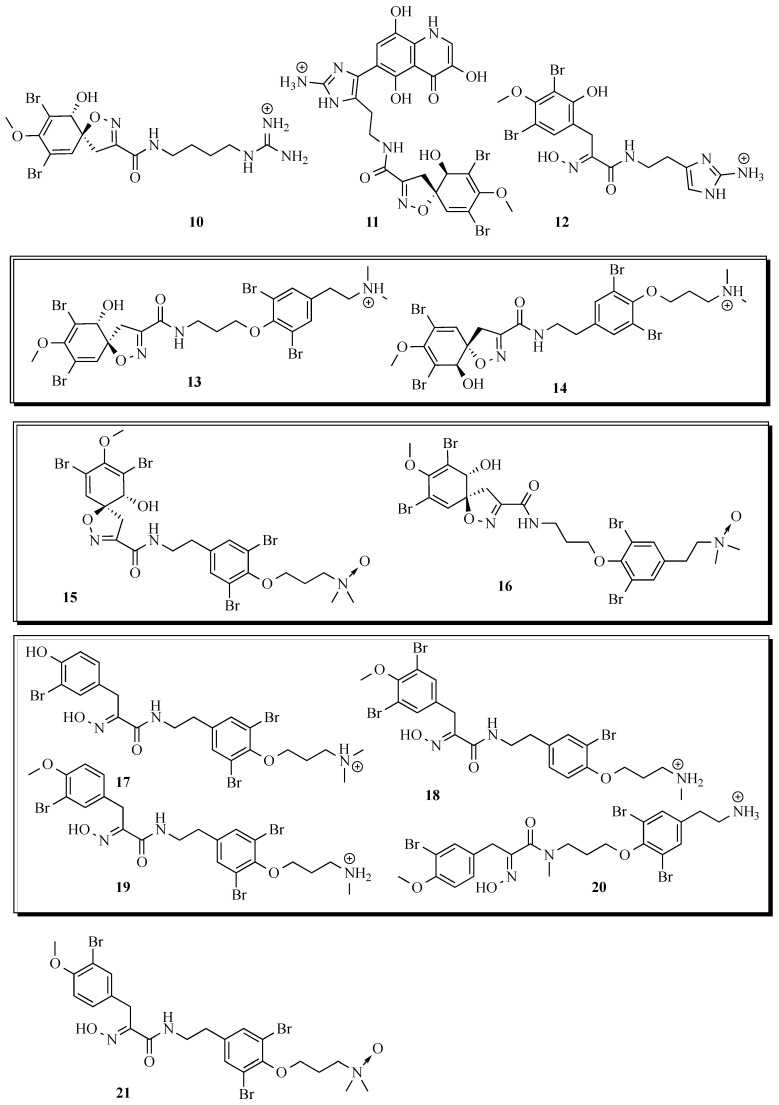
Bromotyrosine derivatives tentatively identified from the ethyl acetate extracts of *P. purpurea* and *T. corticalis* based on UV and HR-MS analysis. The boxed structures are possible isomeric forms present. Stereochemistry shown as reported in the literature.

Returning to the apparent lack of similarity between the metabolites positively identified from the sponge and those tentatively identified from *T. corticalis*, firstly it should be noted that the major difference is that many compounds found at high concentrations in the sponge were not found in the mollusc. This is, however, typically what is observed with other spongiverous molluscs such as nudibranchs, for example [25]. Next, four of the eight major compounds from the sponge were detected in the mollusc, albeit at a lower level and two of the minor/trace metabolites from the sponge were also detected in the mollusc at much higher relative concentrations. The notable difference is the high concentrations of purealidin T and purpuramine J, which were not observed in the sponge extract at all despite a careful search. However, purealidin T is the *N*-oxide of purealidin Q and purpuramine J is the *N*-oxide of aplysamine 2, a major alkaloid from the sponge. Taken together these observations suggest that the sponge metabolites are certainly accumulated on a selective basis (purealidin P/Q) but also that the mollusc might be modifying the sponge metabolites (purealidin T, purpuramine J) to *N*-oxides. Many cases of modification of sponge metabolites by predatory molluscs have previously been reported [57]. Certainly, the major metabolites isolated from *T. corticalis* are *N*-oxides closely related to compounds found in the sponge (Figure 7).

## 3. Experimental Section

### 3.1. General Experimental Procedures

^1^H NMR and ^13^C NMR spectra were recorded in 5 mm Pyrex tubes on a Bruker Avance DPX-400 400 MHz or DRX-600K 600 MHz spectrometer. All spectra were obtained at 25 °C, processed using Bruker Topspin 1.3 and referenced to residual solvent (DMSO-*d*_6_ 2.50/39.52 ppm). Infrared spectra were taken on a Perkin Elmer paragon 1000PC FTIR spectrometer, or Nicolet iS10 FT-IR Spectrometer (Thermo Scientific, Scorsby, Australia). UV measurements were performed on a NanoDrop 2000 UV-Vis-Spectrophotometer (Thermo Scientific, Scorsby, Australia). The specific optical rotation was measured on a Jasco P-1010 Polarimeter (Jasco, Tokyo, Japan). Low resolution mass spectrometry was performed by electrospray ionisation (ESI) MS in positive polarity mode on a Shimadzu LC-20A prominence system coupled to a LCMS-2010 EV mass spectrometer using LCMSsolution 3.21 software. LC-MS experiments were carried out on a Gemini C18 column (Phenomenex, Sydney, Australia) 150 × 2 mm, 110Å, 3 µm. High resolution-LC-MS experiments were carried out on a Thermo Orbitrap Elite mass spectrometer with Easy Spray source. The samples were loaded on a C18 trap column (Acclaim, Pepmap 100, 75 µm × 2 cm, nanoviper, C18, 3 µm, 100 Å) and switched online to the analytical column (Thermo, Easy Spray column, PepMap C18 column RSLC, C18, 2 µm, 100 Å, 50 µm × 15 cm) after desalting. Samples were analysed in positive polarity mode using XCalibur2.2 software.

HPLC separations were achieved on a Shimadzu 10AD-VP system running Class-VP 7.4 SP1 software. Analytical and preparative HPLC were performed on Gemini C18 HPLC columns (Phenomenex, Sydney, Australia): Gemini-NX C18 250 × 4.6 mm, 110Å, 5 µm and Gemini-NX C18 150 × 21.2 mm, 110Å, 5 µm respectively. TLC was performed with Merck Kieselgel 60 F_254_ plates with viewing under ultraviolet light (254 nm and 365 nm) and/or by heating, after treatment with 10% phosphomolybdic acid in ethanol. Flash column chromatography was performed on silica gel (60 Å 0.040–0.063 mm, 230–400 mesh ASTM from Merck).

### 3.2. Animal Material

The sponge (654 g) was collected by hand at Jervis Bay, NSW, Australia (S 35°08′07″, E 150°43′33″, at 1–4 m depth) under New South Wales Department of Primary Industries collecting permitNo. P08/0006-1.1. The samples were frozen upon collection and stored at −20 °C. It was identified as *Pseudoceratina purpurea* Carter (phylum Porifera, class Demospongiae, order Verongida, family Aplysinellidae). A voucher specimen (JB-S07) was prepared according to Hooper [58] and deposited at the Department of Chemistry and Biomolecular Sciences, Macquarie University. The opisthobranch *Tylodina corticalis* (Tate) was found feeding on the sponge *Pseudoceratina purpurea*. One individual was separated from the sponge, frozen at −20 °C in the field and stored at −80 °C in the laboratory.

### 3.3. Bioassays

Detailed descriptions of the bioassay-guided fractionation, as well as protocols for the disc diffusion and MTT assay can be found in the Supplementary Information.

### 3.4. Extraction and Isolation

The frozen sponge (654 g) was homogenised in distilled ethanol (4 × 1000 mL) and the combined ethanol extracts filtered through celite and evaporated to approximately 1000 mL. An aliquot (20 mL) was, freeze dried and stored at −80 °C. The remaining solution was partitioned against petroleum ether (3 × 500 mL), ethyl acetate (5 × 500 mL), and 1-butanol (3 × 200 mL) to yield 0.9076 g, 6.5420 g, and 0.2241 g of crude extracts respectively. The crude ethyl acetate extract (3.5 g) was subjected to gel permeation chromatography (Sephadex LH-20; 1:1 chloroform:methanol) and fractions 9–22, containing all the natural products were combined. The resulting dark residue (1.66 g) was purified by HPLC (preparative C18 column, 9.99 mL/min, gradient from 18% to 40% acetonitrile:water:0.05% TFA over 40 min).

(**+**)**-Ceratinadin D** (**1**): Unstable brown amorphous solid (4.5 mg; 0.011% of wet weight). ${\left[\text{α}\right]}_{\text{D}}^{20}$ +52° (*c* 0.45, MeOH). λ_max_ (MeOH) 234, 276, 337 nm. ν_max_ (neat film) 3420 (m), 3380 (m), 3290 (m), 1677 (s), 1432 (s), 1202 (d), 1135 (s), 1047 (s), 1025 (br), 802 (s), 764 (s), 722 (s) cm^−1^. ^1^H-NMR (600 MHz, DMSO-*d*_6_) δ 14.3 (bs, H-12′), 12.15 (bs, H-23), 11.93 (bs, H-21), 11.76 (d, *J* = 6.4 Hz, H-1′), 10.25 (bs, H-13′), 8.93 (bs, H-11′), 8.51 (t, *J* = 5.8 Hz, H-16), 7.28 (bs, (H-25)_2_), 7.64 (d, *J* = 6.4 Hz, H-2′), 6.83 (s, H-8′), 6.54 (s, H-5), 6.36 (d, *J* = 7.9 Hz, H-9), 3.90 (d, *J* = 7.6 Hz, H-1), 3.63 (s, OMe), 3.59 (d, *J* = 3.6 Hz, H-10a), 3.15 (d, *J* = 3.6 Hz, H-10b), 3.13 (m, (H-17)_2_), 2.51 (s, (H-19)_2_), 1.73 (m, (H-18)_2_). ^13^C-NMR (150 MHz, DMSO-*d*_6_) δ 173.2 (C-4′), 158.9 (C-14), 154.5 (C-11), 149.6 (C-6′), 147.2 (C-3), 146.6 (C-22), 139.9 (C-3′), 137.4 (C-9′), 131.2 (C-5), 128.9 (C-10′), 124.2 (C-2′), 122.4 (C-20), 120.9 (C-2), 117.8 (C-24), 113.7 (C-8′), 113.1 (C-4), 112.6 (C-5′), 103.7 (c-7’), 90.1 (C-6), 73.6 (C-1), 59.7 (C-8), 39.0 (C-10), 38.2 (C-17), 28.1 (C-18), 21.6 (C-19). Mass spectrum (ESI+) *m/z*: isotopic cluster 695:697:699 (in ratio 1:2:1). (HRESI+) Found *m/z*: 695.0067, C_25_H_25_N_6_O_8_^79^Br_2_ requires 695.0101.

**Aplysamine 8** (**2**): Light yellow solid (0.9 mg; 0.002% of wet weight). λ_max_ (MeOH) 209, 278 nm. ν_max_ (neat film) 3572 (m), 3187 (m), 3061 (m), 2930 (m), 1721 (m), 1685 (s) 1672 (m), 1642 (m), 1631 (m), 1546 (m), 1422 (s), 1258 (s), 1201 (br), 1001 (s), 835 (s), 734 (s), 721 (s) cm^−1^. ^1^H-NMR (400 MHz, DMSO-*d*_6_) δ 12.02 (s, H-10), 8.12 (t, *J* = 6.0 Hz, H-13), 7.81 (bs, (H-24)_2_), 7.55 (s, (H-20)_2_), 7.44 (s, (H-2)_2_), 3.88 (t, *J* = 6.4 Hz, (H-16)_2_), 3.76 (s, (H-7)_2_), 3.75 (s, OMe), 3.38 (m, (H-14)_2_), 3.05 (m, (H-23)_2_), 2.81 (t, *J* = 7.4 Hz, (H-22)_2_), 1.96 (m, (H-15)_2_). ^13^C-NMR (100 MHz, DMSO-*d*_6_) δ 163.0 (C-11), 151.8 (C-4), 151.3 (C-18), 151.0 (C-8), 136.8 (C-21), 136.3 (C-1), 133.2 ((C-20)_2_), 132.9 ((C-2)_2_), 117.6 ((C-19)_2_), 117.1 ((C-3)_2_), 71.3 (C-16), 60.4 (OMe), 27.9 (C-7), 39.4 (C-23), 36.2 (C-14), 31.5 (C-22), 29.6 (C-15). Mass spectrum (ESI+) *m/z*: isotopic cluster 698:700:702:704:706 (in ratio 1:4:6:4:1). (HRESI+) Found 697.8527, C_21_H_24_^79^Br_4_N_3_O_4_ requires 697.8500.

(**−**)**-Pseudoceratinin A** (**3**): [45] Unstable light yellow solid (15.4 mg; 0.038% of wet weight). ${\left[\text{α}\right]}_{\text{D}}^{20}$ −154° (*c* 0.5, MeOH) Lit. −158°. ^1^H-NMR (600 MHz, DMSO-*d*_6_) δ 12.10 (s, NH-13), 11.80 (s, NH-12), 8.60 (t, *J* = 8.6 Hz, NH-9), 7.40 (NH_2_-14), 6.59 (s, H-13), 6.55 (bs, C1-OH), 6.54 (s, H-5), 3.89 (s, H-1), 3.62 (s, OMe), 3.59 (d, *J* = 18.2 Hz, H-7a), 3.35 (q, *J* = 6.4 Hz, (H-10)_2_), 3.17 (d, *J* = 18.1 Hz, H-7b), 2.59 (t, *J* = 6.5 Hz, (H-11)_2_). ^13^C-NMR (150 MHz, DMSO-*d*_6_) δ 159.5 (C-9), 154.9 (C-8), 147.6 (C-3), 147.5 (C-14), 131.7 (C-5), 124.7 (C-12), 121.3 (C-2), 113.6 (C-4), 109.9 (C-13), 90.7 (C-6), 74.1 (C-1), 60.1 (MeO), 39.5 (C-7), 37.8 (C-10), 24.8 (C-11). Mass spectrum (ESI+) *m/z*: isotopic cluster 490:492:494 (in ratio 1:2:1).

(**+**)**-Aerophobin 2** (**4**): [46] Off-white solid (9.1 mg; 0.022% of wet weight). ${\left[\text{α}\right]}_{\text{D}}^{20}$ +128° (*c* 0.55, MeOH), Lit. [46] +139°. ^1^H-NMR (600 MHz, DMSO-*d*_6_) δ 12.11 (s, NH-14), 11.70 (s, NH-13), 8.57 (t, *J* = 5.9 Hz, NH-9), 7.37 (s, NH_2_-15), 6.57 (s, H-14), 6.56 (s, H-5), 3.90 (s, H-1), 3.63 (s, OMe), 3.60 (d, *J* = 17.8 Hz, H-7a), 3.18 (d, *J* = 17.8 Hz, H-7b), 3.16 (q, *J* = 6.6 Hz, (H-10)_2_), 2.39 (t, *J* = 7.4 Hz, (H-12)_2_), 1.69 (m, (H-11)_2_). ^13^C-NMR (150 MHz, DMSO-*d*_6_) δ 159.5 (C-9), 155.0 (C-8), 147.6 (C-3), 147.3 (C-15), 131.7 (C-5), 126.8 (C-13), 121.3 (C-2), 113.6 (C-4), 109.3 (C-14), 90.7 (C-6), 74.1 (C-1), 60.1 (MeO), 39.8 (C-7), 38.5 (C-10), 27.8 (C-11), 22.0 (C-12). Mass spectrum (ESI+) *m/z*: isotopic cluster 504:506:508 (in ratio 1:2:1).

(**−**)**-Hexadellin A** (**5**): [47] Slight-pink solid (8.1 mg; 0.020% of wet weight). ${\left[\text{α}\right]}_{\text{D}}^{20}$ −19° (*c* 0.5, MeOH). ^1^H-NMR (400 MHz, DMSO-*d*_6_) δ 8.55 (t, *J* = 5.6 Hz, H-10), 7.82 (bs, (H-20)_2_), 7.57 (bs, (H-16)_2_), 6.56 (bs, H-5), 6.36 (bs, C1-OH), 3.94 (t, *J* = 6.2 Hz, (H-13)_2_), 3.90 (s, H-1), 3.62 (s, OMe), 3.58 (d, *J* = 18.2 Hz, H-7a), 3.19 (d, *J* = 18.2 Hz, H-7b), 3.38 (m, H-11), 3.05 (m, (H-19)_2_), 2.81 (t, *J* = 7.2 Hz, (H-18)_2_), 1.98 (m, (H-12)_2_). ^13^C-NMR (100 MHz, DMSO-*d*_6_) δ 159.4 (C-9), 155.0 (C-8), 151.7 (C-14), 147.6 (C-3), 137.3 (C-17), 133.7 (C-16), 131.7 (C-5), 121.3 (C-2), 118.1 (C-15), 113.6 (C-4), 90.7 (C-6), 74.1 (C-1), 71.7 (C-13), 60.1 (OMe), 39.0 (C-7), 39.0 (C-19), 36.7 (C-11), 32.0 (C-18), 29.9 (C-12). Mass spectrum (ESI+) *m/z*: isotopic cluster 714:716:718:720:722 (in ratio 1:4:6:4:1).

**Aplysamine 2** (**6**): [48] Light brown solid (9.0 mg; 0.022% of wet weight). ^1^H-NMR (400 MHz, DMSO-*d*_6_) δ 11.90 (s, N-OH), 8.05 (t, *J* = 6.0 Hz, NH-9), 7.48 (s, H-13 and H-17), 7.37 (d, *J* = 2.0 Hz, H-2), 7.11 (dd, *J* = 8.5 Hz, 2.1 Hz, H-6), 6.97 (d, *J* = 8.5 Hz, H-5), 3.96 (t, *J* = 5.9 Hz, (H-18)_2_), 3.78 (s, OMe), 3.70 (s, (H-7)_2_), 3.35 (m, (H-10)_2_), 3.34 (m, (H-20)_2_), 2.83 (s, (NMe_2_), 2.72 (t, *J* = 7.0 Hz, H-11), 2.18 (m, (H-19)_2_). ^13^C-NMR (100 MHz, DMSO-*d*_6_) δ 163.3 (C-9), 153.0 (C-4), 151.8 (C-8), 150.2 (C-15), 139.4 (C-12), 133.1 (C-13 and C-17), 133.0 (C-2), 129.2 (C-6), 117.2 (C-14 and C-16), 113.8 (C-5), 112.6 (C-3), 110.1 (C-1), 70.2 (C-18), 56.2 (OMe), 42.4 (NMe_2_), 39.6 (C-10), 39.6 (C-20), 33.4 (C-11), 27.8 (C-7), 24.8 (C-19). Mass spectrum (ESI+) *m/z*: isotopic cluster 648:650:652:654 (in ratio 1:3:3:1).

**16-Debromoaplysamine 4** (**7**): [49] Light brown solid (1.6 mg; 0.004% of wet weight). ^1^H-NMR (400 MHz, DMSO-*d*_6_) δ 11.98 (s, N-OH), 8.16 (t, *J* = 5.9 Hz, NH-9), 7.77 (s, NH), 7.47 (s, H-13), 7.45 (s, H-1 and H-5), 7.19 (dd, *J* = 8.4 Hz, 2.1 Hz, H-17), 7.00 (d, *J* = 8.4 Hz, H-16), 3.99 (t, *J* = 6.1 Hz, (H-18)_2_), 3.75 (s, (H-7)_2_), 3.74 (s, (OMe), 3.34 (m, (H-20)_2_), 3.00 (t, *J* = 7.8 Hz, (H-10)_2_), 2.77 (t, *J* = 7.7 Hz, (H-11)_2_), 1.92 (m, (H-19)_2_). ^13^C-NMR (100 MHz, DMSO-*d*_6_) δ 163.3 (C-9), 153.6 (C-15), 151.8 (C-3), 151.1 (C-8), 136.3 (C-6), 133.0 (C-13), 132.8 (C-1, C-5), 131.2 (C-12), 129.2 (C-17), 117.0 (C-2, C-4), 113.7 (C-16), 111.3 (C-14), 66.7 (C-18), 60.2 (OMe), 39.8 (C-10), 36.1 (C-20), 31.7 (C-11), 28.7 (C-19), 27.9 (C-7). Mass spectrum (ESI+) *m/z*: isotopic cluster 620:622:624:626 (in ratio 1:3:3:1).

(**−**)**-Purealin** (**8**): [50] Light brown solid (1.9 mg; 0.005% of wet weight). ${\left[\text{α}\right]}_{\text{D}}^{20}$ −82° (*c* 0.1, MeOH), Lit. [50] −85°. ^1^H-NMR (400 MHz, DMSO-*d*_6_) δ 12.03 (s, N-OH), 11.85 (s, NH-22), 8.57 (bs, NH-9), 8.15 (bs, NH-19), 7.44 (s, H-15 and H-15′), 7.35 (bs, NH_2_-24), 6.57 (d, *J* = 10.6 Hz, H-5), 6.56 (s, H-23), 6.38 (bs, C1-OH), 3.90 (d, *J* = 8.1 Hz, H-1), 3.75 (s, (H-17)_2_), 3.63 (s, (OMe), 3.61 (d, *J* = 18.1 Hz, H-7a), 3.49 (m, (H-12)_2_), 3.38 (m, (H-10)_2_), 3.36 (m, (H-20)_2_), 3.20 (d, *J* = 18.3 Hz, H-7b), 2.59 (m, (H-21)_2_), 1.97 (m, (H-11)_2_). ^13^C-NMR (100 MHz, DMSO-*d*_6_) δ 163.3 (C-19), 158.9 (C-9), 154.5 (C-8), 151.7 (C-13), 151.0 (C-18), 147.1 (C-3), 136.3 (C-16), 132.9 (C-15 and C-15′), 131.3 (C-5), 124.4 (C-22), 120.8 (C-2), 117.1 (C-14 and C-14′), 113.1 (C-4), 109.2 (C-23), 90.2 (C-6), 73.6 (C-1), 69.8 (C-12), 59.4 (OMe), 39.4 (C-7), 37.3 (C-20), 36.2 (C-10), 29.4 (C-11), 27.9 (C-17), 24.5 (C-21). Mass spectrum (ESI+) *m/z*: isotopic cluster 880:882:884:886:888 (in ratio 1:4:6:4:1).

The opisthobranch *Tylodina corticalis* was extracted by soaking overnight in ethanol (5 × 50 mL) at −20 °C. The combined extracts (327.6 mg) were partitioned against petroleum ether (3 × 30 mL), ethyl acetate (4 × 30 mL) and 1-butanol (5 × 30 mL) to yield 40.1, 12.0 mg, and 95.6 mg of crude extract respectively. An aliquot (1 mg) of the ethyl acetate extract was dissolved in 10% acetonitrile/water/0.1% formic acid (1 mL) and analysed by low resolution-LC-MS (Gemini C18 column, 0.2 mL/min, gradient from 10% to 95% acetonitrile/water/0.1% formic acid over 35 min. For HR-LC-MS analysis 2 μL of samples (20 µg/mL) were loaded on a C18 trap column. After desalting, samples were eluted over 30 min from 5% acetonitrile/0.1% formic acid to 90% acetonitrile/0.1% formic acid at 300 nL/min.

## 4. Conclusions

This is the first report of two previously unknown brominated tyrosine derivatives (**1** and **2**) including a full structure analysis of these and six other compounds from *Pseudoceratina purpurea*. The antimicrobial activity of the isolated compounds was assessed against common human pathogens in preliminary assays and showed moderate activity for compound **2** against *S. aureus*. Seven further compounds found in the sponge or its predator, were tentatively assigned by UV and high resolution MS data.

## Supplementary Information

Supplementary Information including details on the bioassays, biological material, HRMS and NMR data are available upon request.
